# The Leucine-rich Pentatricopeptide Repeat-containing Protein (LRPPRC) Does Not Activate Transcription in Mammalian Mitochondria*[Fn FN2]

**DOI:** 10.1074/jbc.M113.471649

**Published:** 2013-04-18

**Authors:** Julia Harmel, Benedetta Ruzzenente, Mügen Terzioglu, Henrik Spåhr, Maria Falkenberg, Nils-Göran Larsson

**Affiliations:** From the ‡ Department of Mitochondrial Biology, Max Planck Institute for Biology of Ageing, D-50931 Cologne, Germany,; the § Institute of Biomedicine, University of Gothenburg, SE-405 30 Gothenburg, Sweden, and; the ¶ Department of Laboratory Medicine, Karolinska Institutet, SE-171 77 Stockholm, Sweden

**Keywords:** Mitochondria, Mitochondrial Diseases, Mitochondrial DNA, Mouse Genetics, mRNA, LRPPRC, Mitochondrial RNA Polymerase, Mitochondrial Transcription, Post-transcriptional Regulation

## Abstract

Regulation of mtDNA expression is critical for controlling oxidative phosphorylation capacity and has been reported to occur at several different levels in mammalian mitochondria. LRPPRC (leucine-rich pentatricopeptide repeat-containing protein) has a key role in this regulation and acts at the post-transcriptional level to stabilize mitochondrial mRNAs, to promote mitochondrial mRNA polyadenylation, and to coordinate mitochondrial translation. However, recent studies have suggested that LRPPRC may have an additional intramitochondrial role by directly interacting with the mitochondrial RNA polymerase POLRMT to stimulate mtDNA transcription. In this study, we have further examined the intramitochondrial roles for LRPPRC by creating bacterial artificial chromosome transgenic mice with moderately increased LRPPRC expression and heterozygous *Lrpprc* knock-out mice with moderately decreased LRPPRC expression. Variation of LRPPRC levels in mice *in vivo*, occurring within a predicted normal physiological range, strongly affected the levels of an unprocessed mitochondrial precursor transcript (ND5-cytochrome *b*) but had no effect on steady-state levels of mitochondrial transcripts or *de novo* transcription of mtDNA. We further assessed the role of LRPPRC in mitochondrial transcription by performing size exclusion chromatography and immunoprecipitation experiments in human cell lines and mice, but we found no interaction between LRPPRC and POLRMT. Furthermore, addition of purified LRPPRC to a recombinant human *in vitro* transcription system did not activate mtDNA transcription. On the basis of these data, we conclude that LRPPRC does not directly regulate mtDNA transcription but rather acts as a post-transcriptional regulator of mammalian mtDNA expression.

## Teaser

**Background:** LRPPRC regulates mitochondrial mRNA stability and was recently reported to also stimulate mammalian mtDNA transcription.

**Results:** Altered expression of LRPPRC in mice *in vivo* or addition of LRPPRC to a recombinant transcription system *in vitro* does not affect mtDNA transcription.

**Conclusion:** LRPPRC is not a mitochondrial transcriptional activator.

**Significance:** LRPPRC is an essential post-transcriptional regulator of metazoan mtDNA expression.

## Introduction

Members of the pentatricopeptide repeat (PPR)^2^ protein family play important roles in mitochondrial RNA metabolism in metazoans, plants, and yeast (1). They are RNA-binding proteins characterized by a 35-amino acid long motif, which can be repeated between 2 and 30 times. There are several hundred known PPR protein members in plants (localized to chloroplasts or mitochondria) that have been shown to have important roles in RNA editing, RNA stability, guidance of RNA modification, and initiation of translation (2, 3). In contrast to plants, mammals contain only seven PPR proteins, all localized mainly to mitochondria, with roles in RNA metabolism. The mammalian PPR proteins include POLRMT (4), PTCD1–3 (pentatricopeptide repeat domain-containing proteins 1–3) (5–7), MRPS27 (mitochondrial ribosomal protein S27) (8), MRPP3 (mitochondrial ribonuclease P protein 3) (9), and LRPPRC (leucine-rich pentatricopeptide repeat-containing protein) (10), which have been reported to play different roles in transcription, processing of polycistronic RNAs, biogenesis of the small ribosomal subunit, mRNA stability, and mRNA polyadenylation.

LRPPRC forms a complex with SLIRP (stem-loop-interacting RNA-binding protein) and controls mRNA stability, mRNA polyadenylation, and coordination of translation (11–13). A recessive mutation creates an A354V amino acid substitution in LRPPRC that causes a neurodegenerative disease called Leigh syndrome French Canadian variant, which is characterized by cytochrome *c* oxidase deficiency, decreased mitochondrial mRNA levels, and reduced mitochondrial translation in liver and brain (11, 14). There are many reports that describe roles for LRPPRC in RNA transport from the nucleus to the cytoplasm (10), in regulation of cytoplasmic translation (15), and in nuclear transcription (16). However, the main part of LRPPRC is found in mitochondria (17), and RNAi knockdown of LRPPRC expression in cell lines (11) and conditional knock-out of *Lrpprc* in mice (12) have both shown a strong reduction in mtDNA expression. Homozygous knock-out of *Lrpprc* in mice is embryonic lethal, and tissue-specific disruption in heart creates a strong mitochondrial phenotype with decreased steady-state levels of mRNAs, defective polyadenylation, impaired coordination of translation, and cytochrome *c* oxidase deficiency (12). LRPPRC is thus important for post-transcriptional regulation of mtDNA expression in mammals (12). Forced expression of LRPPRC in mouse liver has been reported to cause cristae compaction and stimulation of oxidative phosphorylation (18). This effect has been attributed to a role for LRPPRC as a transcriptional activator, mediated by direct interactions with POLRMT (18).

In this study, we have further characterized a putative role for LRPPRC in mitochondrial transcription by manipulating the *in vivo* expression of LRPPRC in mice, by biochemical fractionation of mitochondrial extracts, and by performing *in vitro* transcription reactions. We report a novel role for LRPPRC in mitochondrial RNA processing, but the *in vivo* and *in vitro* findings we present here do not support the hypothesis that LRPPRC also stimulates mtDNA transcription. LRPPRC rather seems to have a specific role in post-transcriptional regulation of mtDNA expression.

## EXPERIMENTAL PROCEDURES

### 

#### 

##### Generation of Lrpprc-overexpressing and Heterozygous Lrpprc Knock-out Mice

A bacterial artificial chromosome (BAC) clone of 241 kb (RP24–100M10) containing the whole mouse *Lrpprc* gene was obtained from the Children's Hospital Oakland Research Institute BACPAC Resources Center. The BAC was modified by RecE and RecT protein mediated recombination to allow discrimination between transcripts expressed from the endogenous *Lrpprc* gene and the introduced BAC clone. A silent mutation that did not alter the encoded amino acid but did eliminate a BglII site was introduced in exon 3. The modified BAC was purified by cesium chloride gradient centrifugation and injected into the pronuclei of fertilized oocytes. Founders (+/BAC-LRPPRC) were identified by PCR and restriction enzyme analysis of genomic DNA to detect loss of the BglII site in the *Lrpprc* gene. Tail DNA from offspring was genotyped for the presence of the BAC transgene by analyzing 100 ng of tail DNA with the GoTaq PCR reaction kit (Promega) according to the manufacturer's instruction by adding forward primer 5′-AAATTTGTTTCTCTTTGGACTTATTAGTTT-3′ and reverse primer 5′-TTATAATACTTATGTGAAGAACACAGTGGA-3′ (0.5 pmol each) for PCR with an initial denaturation for 3 min at 95 °C, followed by 35 cycles for 30 s at 95 °C, 30 s at 53 °C, and 45 s at 73 °C. The reaction was ended with extension for 5 min at 72 °C. Breeding and genotyping of heterozygous *Lrpprc* knock-out and *Lrpprc*-FLAG mice were performed as described previously (12). Mice were maintained on an inbred C57BL/6N background.

##### RNA Isolation and Northern Blot Analysis

RNA from mouse heart and liver was extracted with Lysing Matrix D tubes (MP Biomedicals) and the ToTALLY RNA kit (Ambion) following the manufacturers' instructions. The RNA concentration was measured with a NanoDrop 2000c spectrophotometer (Peqlab), and 2 μg of total RNA was separated in a 1.2% agarose gel containing formaldehyde and thereafter transferred to Hybond^TM^-N^+^ nylon membranes (Amersham Biosciences) by Northern blotting. The membranes were stripped before rehybridization with another probe. The efficiency of the stripping procedure was documented by autoradiography. All mitochondrial tRNAs have essentially the same size and therefore migrate together in agarose gels. Gel artifacts in the region where the tRNAs are located will therefore be reiterated if the same blot is rehybridized to detect different tRNAs.

##### DNA Isolation and Southern Blot Analysis

Genomic DNA from mouse heart and liver was extracted with Puregene® Core Kit A (Qiagen) following the instructions of the manufacturer. Total DNA (10 μg) from liver tissue of 10–12-week-old animals was used as described previously (19). Wet transfer was performed in 20× SSC overnight on a Hybond^TM^-N^+^ nylon membrane. A plasmid (pAM1) containing cloned mouse mtDNA was used to detect mtDNA. A plasmid containing the nucleus-encoded 18 S rRNA gene was used to detect cytoplasmic 18 S rRNA as a loading control.

##### Western Blot Analysis

Isolated mitochondria (20 μg) from heart, liver, kidney, and muscle were pelleted and resuspended in SDS/Laemmli buffer. Protein concentration was determined with the Bradford method (Bio-Rad). Samples were run in 4–12% NuPAGE gels (Invitrogen) at 200 V for 50 min. Western blot analysis was performed using standard protocols. Mouse monoclonal antibodies detecting nucleus-encoded subunits of mouse complex I (NDUFA9 subunit; 1:1000; Invitrogen), complex II (SDHA subunit, 1:1000; Invitrogen), complex III (UQCRC2 subunit, 1:1000; Invitrogen), complex IV (COX1 and COX3 subunits; 1:1000; Invitrogen), and complex V (ATP5A1 subunit); MitoProfile total oxidative phosphorylation antibody mixture (1:1000; MitoSciences); and porin (1:1000; MitoSciences) were used for analysis of levels of mitochondrial respiratory chain complexes. Mouse and human FLAG-tagged LRPPRC proteins were detected with anti-FLAG monoclonal antibody M2 (1:250; Sigma). Human POLRMT and SLIRP were detected with polyclonal antibodies (1:1000; Abcam). Rabbit polyclonal antisera generated in-house were used to detect mouse LRPPRC (1:250), mouse TFAM (transcription factor A, mitochondrial; 1:500), mouse POLRMT (1:50), and mouse TFB2M (transcription factor B2, mitochondrial; 1:50).

##### De Novo Transcription Assays

Isolated mitochondria (2 mg) from heart and liver tissue were pelleted and resuspended in 500 μl of transcription buffer containing 25 mm sucrose, 75 mm sorbitol, 100 mm KCl, 10 mm K_2_HPO_4_, 50 mm EDTA, 5 mm MgCl_2_, 1 mm ADP, 10 mm glutamate, 2.5 mm malate, and 10 mm Tris-HCl (pH 7.4) with 1 mg of BSA/ml. The mitochondrial suspension containing 50 μCi of [α-^32^P]UTP (Amersham Biosciences) was incubated by rotating the mixture for 1 h at 37 °C. After the incubation, the mitochondria were pelleted and washed twice with resuspension buffer containing 10% glycerol, 10 mm Tris-HCl (pH 6.8), and 0.15 mm MgCl_2_. Mitochondrial RNA was isolated from the final pellet using the ToTALLY RNA kit and resuspended in 30–50 μl of glyoxal loading buffer with dye (Ambion). Samples were separated in 1.2% agarose gel containing formaldehyde at 120 V for 2 h. Additional procedures were as described under “RNA Isolation and Northern Blot Analysis.”

##### Quantitative PCR

Total RNA from mouse liver was extracted using the ToTALLY RNA kit. Reverse transcription and quantitative RT-PCR were performed using the High Capacity RNA-to-cDNA kit (Applied Biosystems) and TaqMan® 2× Universal PCR Master Mix, No AmpErase® UNG (Applied Biosystems). The following custom-made TaqMan probes against mouse mitochondrial transcripts were obtained from Applied Biosystems: cytochrome *b* (Cytb), ND6, and COXI. 18 S rRNA was used as a probe to detect this nuclear transcript.

##### Immunoprecipitation

Mitochondria from stably transfected HeLa Tet-On cell lines expressing human LRPPRC-FLAG and transgenic mice expressing mouse LRPPRC-FLAG in a homozygous *Lrpprc* knock-out background (genotype *Lrpprc*^−/−^, +/BAC-*LRPPRC*-FLAG) were used for immunoprecipitation (12). Human or mouse mitochondria were isolated by differential centrifugation in buffer A (320 mm sucrose, 1 mm EDTA, and 10 mm Tris-HCl (pH 7.4)) containing 1× Complete protease inhibitor mixture (Roche Applied Science). Mitochondria (1 mg) were incubated in lysis buffer B (50 mm Tris-HCl (pH 7.4), 1 mm EDTA, 150 mm NaCl, 5% glycerol, and 0.5% Triton X-11) and 1× Complete protease inhibitor mixture for 20 min on ice, followed by centrifugation at 13,000 × *g* for 45 min at 4 °C. Next, the lysate was incubated with anti-FLAG M2 affinity gel (Sigma), and protein partners were purified according to the recommendations of the manufacturer.

##### Size Exclusion Chromatography

Size exclusion chromatography was performed as described previously (12) with some modifications. Human mitochondria were isolated from HeLa cells by differential centrifugation in isolation buffer A containing 1× Complete protease inhibitor mixture. Mitochondria were lysed at a concentration of 5 mg/ml in lysis buffer B and 1× Complete protease inhibitor mixture for 20 min on ice, followed by centrifugation at 13,000 × *g* for 45 min at 4 °C. Next, 1 mg of the precleared lysate was subjected to size exclusion chromatography on a Superose 6 column (GE Healthcare) that had been pre-equilibrated with lysis buffer B. Fractions of 1 ml were collected, precipitated with TCA, and analyzed by SDS-PAGE and immunoblotting.

##### Recombinant Proteins

For mitochondrial *in vitro* transcription, recombinant human TFAM and TFB2M were expressed and purified from insect cells as described previously (20). POLRMT was expressed and purified from *Escherichia coli* ArcticExpress cells (Stratagene). A DNA fragment encoding LRPPRC fused to a His_6_ tag at the C terminus was cloned into the vector pBacPAK9 (Clontech), and this construct was used to create *Autographa californica* nuclear polyhedrosis recombinant viruses as recommended by the manufacturer. Recombinant human LRPPRC was expressed in Sf9 cells, and whole-cell protein extracts were generated and purified over Ni^2+^-agarose FF (Qiagen) as described (20). LRPPRC was loaded onto a 1-ml HiTrap heparin column (Amersham Biosciences) equilibrated with buffer C (20 mm Tris-HCl (pH 8.0), 0.5 mm EDTA (pH 8.0), 10% glycerol, and 1 mm DTT) containing 0.2 m NaCl. LRPPRC was eluted with a linear gradient (10 ml) of buffer C containing 0.2–1.2 m NaCl, and the peak fractions were diluted three times with buffer C containing 0 m NaCl, followed by further purification on a 1-ml HiTrap SP column (Amersham Biosciences) equilibrated with buffer C containing 0.2 m NaCl. After washing the column with 3 column volumes of buffer C containing 0.2 m NaCl, LRPPRC was eluted with a linear gradient (10 ml) of buffer C containing 0.2–1.2 m NaCl, and the protein peak eluted at 600 mm NaCl. The peak fractions were dialyzed against buffer C containing 0.2 m NaCl. The estimated purity of the purified LRPPRC was at least 95% as estimated using Coomassie Blue-stained SDS-polyacrylamide gels. For absolute quantification, codon-optimized (DNA2.0) DNA encoding the mature form of mouse LRPPRC fused to a His_6_ tag at the N terminus was cloned in the vector pJexpress 401 and heterologously expressed in ArcticExpress(DE3) cells (Stratagene) after induction with 0.2 mm isopropyl 1-thio-β-d-galactopyranoside at 16 °C for 20 h. LRPPRC was purified following the procedure for the MTERF4-NSUN4 complex as described previously (21). Mouse POLRMT, TFB2M, and TFAM were purified as described previously (22).

##### In Vitro Transcription

Plasmid constructs with human mtDNA sequences corresponding to bp 1–741 (light (LSP) and heavy (HSP) strand promoters), 1–477 (LSP), and 499–741 (HSP) were used as templates as described previously (20). *In vitro* transcription reactions contained 100 fmol of the indicated template, 20 mm Tris-HCl (pH 8.0), 10 mm MgCl_2_, 1 mm DTT, 100 μg/ml BSA, 400 μm ATP, 150 μm CTP, 150 μm GTP, 10 μm UTP, 0.2 μm [α-^32^P]UTP (3000 Ci/mmol), 4 units of RNasin (AP-Biotech), 400 fmol of POLRMT, 400 fmol of TFB2M, and 5 pmol of TFAM (15 pmol of TFAM was added when the LSP/HSP template was used). The reaction volume was 25 μl, and the final concentration of NaCl was adjusted to exactly 80 mm NaCl in all reactions. The concentrations of LRPPRC are indicated in the figure legends. Reactions were stopped after 30 min at 32 °C by the addition of 200 μl of stop buffer (10 mm Tris-HCl (pH 8.0), 0.2 m NaCl, 1 mm EDTA, and 0.1 mg/ml glycogen). The samples were treated with 0.5% SDS and 100 μg/ml proteinase K for 45 min at 42 °C and precipitated by the addition of 0.6 ml of ice-cold ethanol. The pellets were dissolved in 10 μl of gel loading buffer (98% formamide, 10 mm EDTA (pH 8.0), 0.025% xylene cyanol FF, and 0.025% bromphenol blue) and heated at 95 °C for 5 min. Transcription reaction products were analyzed in a 6% denaturing polyacrylamide gel with 1× Tris borate/EDTA buffer.

##### Electrophoresis Mobility Shift Assay

The RNA-binding activity of LRPPRC was assayed by EMSA using the HSP (bp 499–741) run-off transcript as a template. A 10× *in vitro* transcription reaction (250 μl) was performed as described above. After 30 min, 2 μl of DNase I (1 unit/μl) was added to the reaction, which was placed on a benchtop at room temperature for 10 min before the radioactive labeled RNA was purified using the RNeasy mini kit (Qiagen). The RNA was eluted in 50 μl of RNase-free water. The RNA-binding reactions were performed in a volume of 20 μl containing 5 μl of the purified RNA, 25 mm Tris-HCl (pH 7.8), 1 mm DTT, 10 mm MgCl_2_, 0.1 mg/ml BSA, 10% glycerol, and different concentrations of LRPPRC as indicated in the figure legends. The reactions were incubated for 20 min on ice before separation on a 4% polyacrylamide gel in 0.5× Tris borate/EDTA buffer for 2 h at 100 V.

##### Blue Native PAGE

Isolated mitochondria (20 μg) were pelleted using the NativePAGE^TM^ Novex® BisTris gel system (Invitrogen) according to the instructions of the manufacturer with sample buffer containing 1% *n*-dodecyl β-d-maltoside. Samples were run on a NativePAGE Novex BisTris gel following the manufacturer's instructions. For the blotting procedure used, see “Western Blot Analysis.”

##### Absolute Quantification of Recombinant Proteins

The molecular weights of recombinant mouse POLRMT, TFAM, TFB2M, and LRPPRC, all without the mitochondrial targeting sequence, were determined as described previously (12, 23, 24). Molarity was calculated according to the molecular weight of the recombinant protein and its obtained concentration.

## RESULTS

### 

#### 

##### Heterozygous Lrpprc Knock-out and Lrpprc-overexpressing Mice Are Fertile and Viable

**FIGURE 1.**
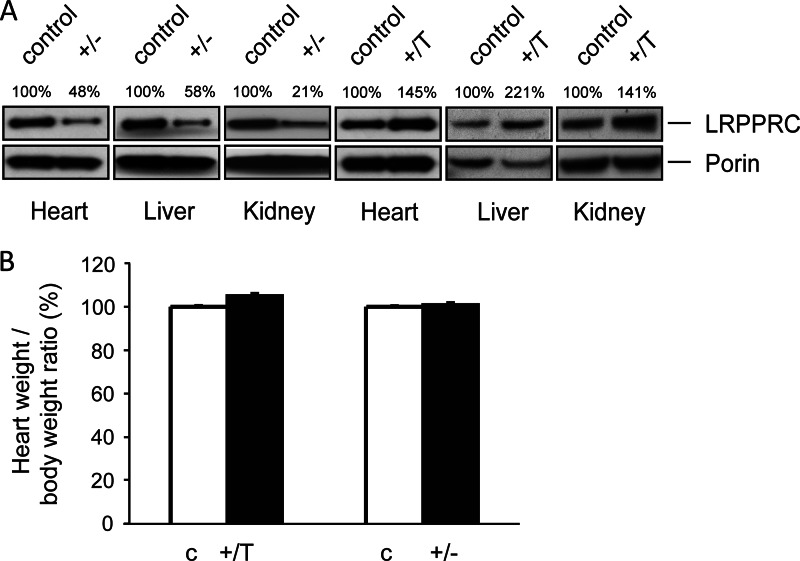
**Heart weight/body weight ratio is not affected by moderately altered levels of LRPPRC.** *A*, Western blot analysis of LRPPRC levels in mitochondrial extracts of heart, liver, and kidney from control, heterozygous *Lrpprc* knock-out (+/−), and *Lrpprc*-overexpressing (+/T) mice at 10 weeks of age. Porin was used as a loading control. The relative levels of LRPPRC are indicated above the lanes. *B*, heart weight/body weight ratios in control (*c*), heterozygous *Lrpprc* knock-out (+/−), and *Lrpprc*-overexpressing (+/T) mice at 10 weeks of age. The number of animals studied was as follows: *n* = 6 (control), *n* = 6 (*Lrpprc*^+/−^), and *n* = 6 (*Lrpprc*^+/T^). *Error bars* indicate S.D.

To obtain additional insights into the *in vivo* function of LRPPRC, we decided to generate mice with moderately decreased or increased LRPPRC expression. *Lrpprc*^+/loxP^ mice were mated to mice expressing Cre recombinase under the control of the β-actin promoter to generate heterozygous *Lrpprc* knock-out (*Lrpprc*^+/−^) mice (12). The *Lrpprc*^+/−^ mice showed decreased LRPPRC protein levels in heart, liver, and kidney, in accordance with a 50% reduction in *Lrpprc* gene dosage (Fig. 1*A*). The *Lrpprc*^+/−^ mice were viable, fertile, and apparently healthy. Loss of LRPPRC in heart causes severe mitochondrial cardiomyopathy in *Lrpprc* conditional knock-out mice (12). We therefore checked the ratio of heart weight to body weight in *Lrpprc*^+/−^ mice, but we found no evidence for cardiomyopathy (Fig. 1*B*). To study the *in vivo* effects of moderately increased LRPPRC expression, we generated BAC transgenic mice with a general moderate increase in LRPPRC expression, as documented by increased LRPPRC protein levels in heart, liver, and kidney (Fig. 1*A*). The transgenic animals (genotype *Lrpprc*^+/T^) were viable, fertile, and apparently healthy, with a normal heart weight/body weight ratio (Fig. 1*B*). Mice with moderately altered expression of LRPPRC, corresponding to a predicted normal physiological range, are thus apparently healthy, with no obvious phenotypes.

##### LRPPRC Does Not Regulate the Amount of mtDNA and Respiratory Chain Complexes

**FIGURE 2.**
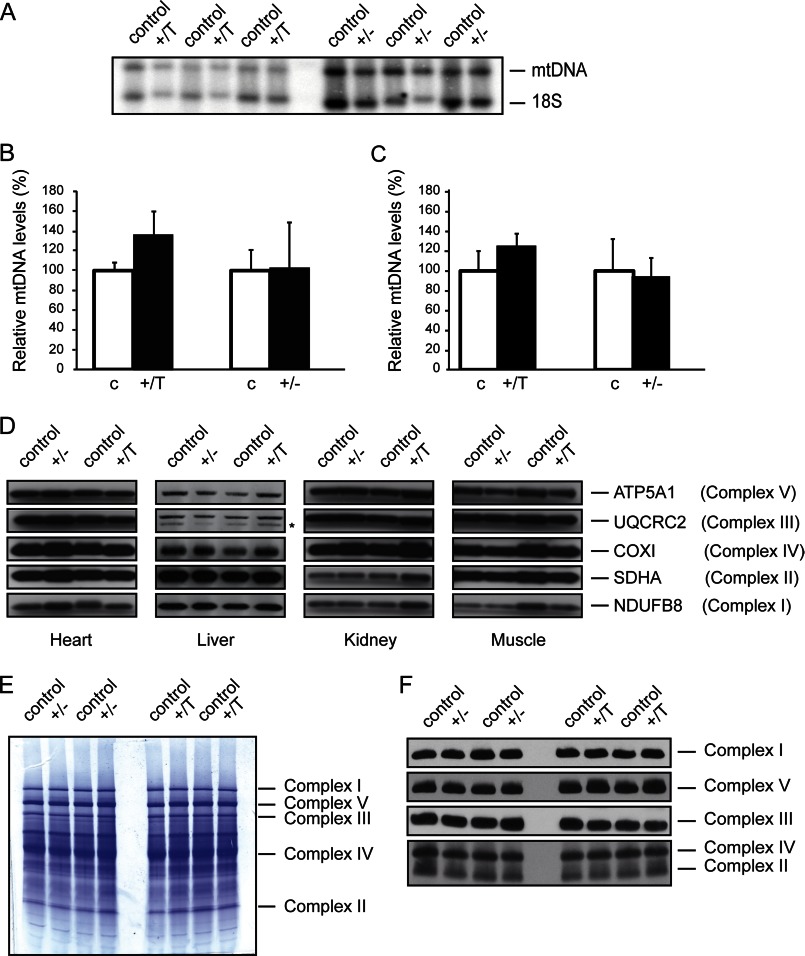
**LRPPRC does not affect oxidative phosphorylation capacity.** *A*, Southern blot analysis of mtDNA levels in liver from control, heterozygous *Lrpprc* knock-out (+/−), and *Lrpprc*-overexpressing (+/T) mice at 10 weeks of age. The number of animals studied was as follows: *n* = 3 (control), *n* = 3 (*Lrpprc*^+/−^), and *n* = 3 (*Lrpprc*^+/T^). The plasmid pAM1, containing cloned mouse mitochondrial DNA, was used as probe to detect mtDNA. The nucleus-encoded 18 S rRNA gene was used as a loading control to detect 18 S rRNA gene. *B*, quantification of mtDNA levels as determined by the Southern blot analysis in *A*. Relative mtDNA levels in control (*c*), heterozygous *Lrpprc* knock-out (+/−), and *Lrpprc*-overexpressing (+/T) mice at 10 weeks of age are shown. The number of animals studied was as follows: *n* = 3 (control), *n* = 3 (*Lrpprc*^+/−^), and *n* = 3 (*Lrpprc*^+/T^). *Error bars* indicate S.D. *C*, quantitative PCR analysis of mtDNA levels in control, heterozygous *Lrpprc* knock-out (+/−), and *Lrpprc*-overexpressing (+/*T*) mice at 10 weeks of age. The number of animals studied was as follows: *n* = 5 (control), *n* = 5 (*Lrpprc*^+/−^), and *n* = 5 (*Lrpprc*^+/T^). *Error bars* indicate S.E.M. (standard error of the mean) *D*, Western blot analysis of subunits of the respiratory chain complexes in mitochondrial extracts of heart, liver, kidney, and muscle from control, heterozygous *Lrpprc* knock-out (+/−), and *Lrpprc*-overexpressing (+/T) mice at 10 weeks of age. Nucleus-encoded complex II was used as a loading control. The *asterisk* indicates a cross-reacting band. *E*, Coomassie Blue staining of a native polyacrylamide gel of mitochondrial extracts from liver of control, heterozygous *Lrpprc* knock-out (+/−), and *Lrpprc*-overexpressing (+/T) mice at 10 weeks of age. *F*, Western blot analysis of levels of respiratory chain complexes in mitochondrial extracts of liver after separation in Blue native polyacrylamide gels. The levels in control, heterozygous *Lrpprc* knock-out (+/−), and *Lrpprc*-overexpressing (+/T) mice are shown. The exclusively nucleus-encoded complex II was used as a loading control.

It has previously been show that LRPPRC is nonessential for mtDNA maintenance (12, 18), despite being identified as a component of the mitochondrial nucleoid (25). We assessed mtDNA levels in mice with moderately decreased and increased LRPPRC expression, but we found no differences in liver from mutant and wild-type mice as determined by Southern blot (Fig. 2, *A* and *B*) and quantitative PCR (Fig. 2*C*) analyses of mtDNA levels. Loss of LRPPRC is known to cause a profound complex IV (cytochrome *c* oxidase) deficiency and to decrease steady-state levels of complexes I and V (11, 12). Moreover, forced expression of LRPPRC in liver has been reported to increase the levels of subunits of the respiratory chain complexes and to remodel mitochondria by increasing cristae density (18). Forced expression of mitochondrial proteins sometimes create artifacts unrelated to the normal physiological function of the studied protein (26). We therefore decided to study *Lrpprc*^+/−^ and *Lrpprc*^+/T^ mice to determine whether moderately altered LRPPRC levels have any effect on steady-state levels of oxidative phosphorylation enzyme complexes. Western blot analysis of respiratory chain subunits showed normal levels of NDUFB8 (complex I), SDHA (complex II), UQCRC2 (complex III), COX1 (complex IV), and ATP5A1 (complex V) in *Lrpprc*^+/−^ and *Lrpprc*^+/T^ mice (Fig. 2*D*). In addition, Blue native PAGE analysis showed normal patterns of assembled respiratory chain complexes and supercomplexes in both mouse models (Fig. 2, *E* and *F*). To summarize, these results show that a moderate increase or decrease in LRPPRC expression has no effect on oxidative phosphorylation capacity *in vivo*.

##### LRPPRC Strongly Influences Levels of the ND5-Cytb Precursor Transcript

**FIGURE 3.**
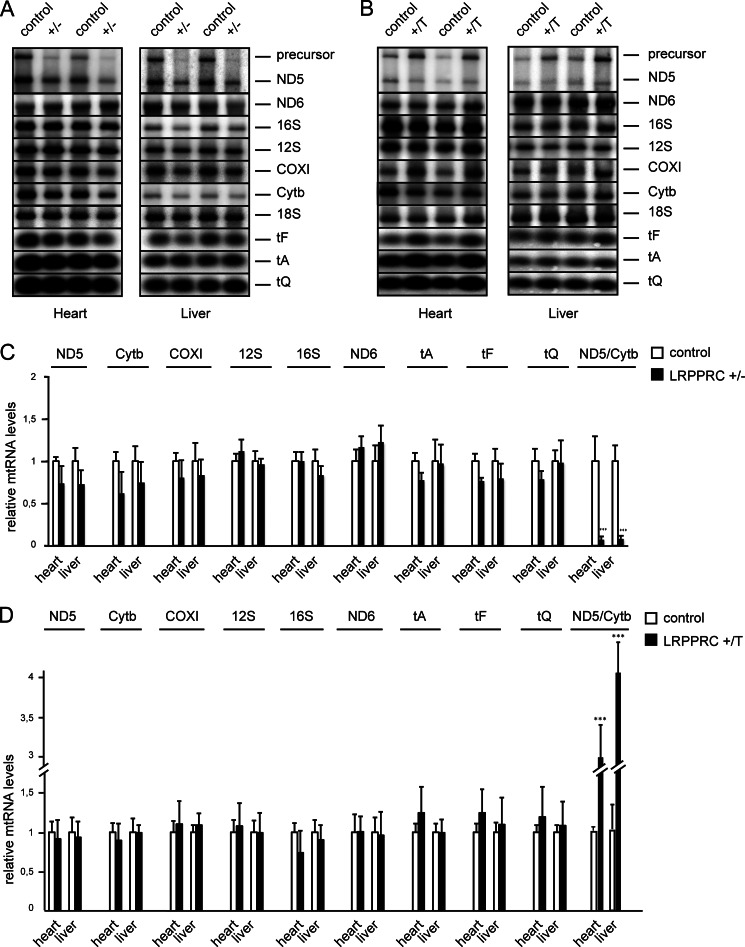
**Steady-state levels of mitochondrial transcripts.** *A*, Northern blot analysis of RNA isolated from heart and liver of control and heterozygous *Lrpprc* knock-out (+/−) mice at 10 weeks of age. A separate autoradiograph is shown for every analyzed transcript. Nucleus-encoded 18 S rRNA (*18S*) was used as a loading control. *B*, Northern blot analysis of RNA isolated from heart and liver of control and *Lrpprc*-overexpressing (+/T) mice at 10 weeks of age. A separate autoradiograph is shown for every analyzed transcript. The nucleus-encoded 18 S rRNA was used as a loading control. The reiterated small artifacts in the panels showing different tRNAs (*tF*, *tA*, and *tQ*) are due to reprobing of a membrane derived from a single gel containing the depicted artifacts. *C*, quantification of steady-state levels of the transcripts from control (+/+; *n* = 5) and heterozygous *Lrpprc* knock-out (+/−; *n* = 5) mice at 10 weeks of age. *Error bars* indicate S.E.M. ***, *p* = 0.001 (Student's *t* test). *D*, quantification of steady-state levels of mitochondrial mRNAs, tRNAs, and rRNAs from control (*n* = 6) and *Lrpprc*-overexpressing (+/T; *n* = 6) mice at 10 weeks of age. *Error bars* indicate S.E.M. ***, *p* = 0.001 (Student's *t* test).

Down-regulation of LRPPRC expression causes severe reduction in the levels of all mRNAs encoded on the heavy strand of mtDNA (11, 12), whereas forced expression of LRPPRC has been reported to lead to accumulation of the same transcripts (12, 27). We further investigated the role of LRPPRC in mtDNA transcription by performing Northern blot analyses of steady-state levels of mitochondrial transcripts in heart and liver from *Lrpprc*^+/−^ and *Lrpprc*^+/T^ mice at 10–12 weeks of age (Fig. 3, *A–D*). The levels of mature mRNAs, rRNAs, and tRNAs were normal in both types of mutant mice (Fig. 3, *A–D*). The finding of normal levels of tRNAs indicates that LRPPRC does not stimulate transcription because we have shown previously that steady-state levels of tRNAs are good indicators of *de novo* transcription activity (12, 28–30). However, we found an RNA-processing defect with a clear change in steady-state levels of a fusion transcript containing the ND5 and Cytb mRNAs, with decreased levels of this precursor RNA species in *Lrpprc*^+/−^ mice (Fig. 3, *A* and *C*) and increased levels in *Lrpprc*^+/T^ mice (Fig. 3, *B* and *D*). The finding of an RNA-processing defect is interesting, as knockdown of the fly homolog to LRPPRC, denoted BSF (bicoid stability factor), also leads to impaired mitochondrial RNA processing (31). Thus, a moderate increase or decrease in the expression of LRPPRC strongly affects the levels of the unprocessed ND5-Cytb transcript, whereas the levels of processed mRNAs, rRNAs, and tRNAs are unaffected.

##### LRPPRC Does Not Activate Mitochondrial Transcription

**FIGURE 4.**
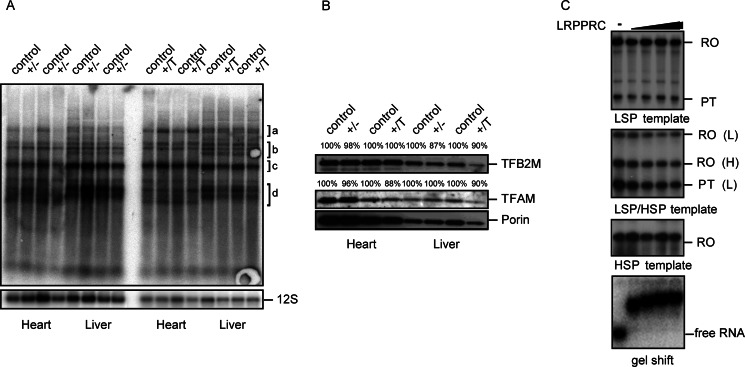
**LRPPRC does not affect mitochondrial transcription.** *A*, *in organello* transcription in heart and liver mitochondria from control, heterozygous *Lrpprc* knock-out (+/−), and *Lrpprc*-overexpressing (+/T) mice. 12 S rRNA (*12S*) was used as a loading control. a–d: chosen areas for quantification, see also supplemental Fig. S1A. *B*, Western blot analysis of the steady-state levels of proteins involved in regulation of mitochondrial transcription (TFAM and TFB2M) in mitochondrial extracts from heart and liver of control, heterozygous *Lrpprc* knock-out (+/−), and *Lrpprc*-overexpressing (+/T) mice at 10–12 weeks of age. Porin was used as a loading control. *C*, *in vitro* transcription was performed with purified recombinant POLRMT, TFB2M, and TFAM and the indicated mtDNA template as described under “Experimental Procedures.” Increasing amounts of LRPPRC (0, 0.4, 0.8, 1.6, and 3.2 pmol) were added where indicated. LSP transcription generated a run-off (*RO*) product as well as a prematurely terminated (*PT*) product. A gel shift assay was used to assess whether recombinant LRPPRC had biological activity and can bind RNA. *L*, light; *H*, heavy.

We isolated mitochondria from heart and liver of *Lrpprc*^+/−^ and *Lrpprc*^+/T^ mice and performed *in organello* transcription reactions. The production of most transcripts was unaffected by the moderately decreased or increased levels of LRPPRC in the *Lrpprc*^+/−^ and *Lrpprc*^+/T^ mice, respectively (Fig. 4*A* and supplemental Fig. S1*A*). However, we observed some changes in the abundance of high molecular weight transcripts (Fig. 4*A*, *bracket a*). The levels of high molecular weight transcripts were decreased in *Lrpprc*^+/−^ mitochondria and increased in *Lrpprc*^+/T^ mitochondria (Fig. 4*A* and supplemental Fig. S1*A*), consistent with the previously observed role for LRPPRC in RNA processing (Fig. 3, *A–D*). There were no changes in the levels of the mitochondrial transcription factors TFAM (32) and TFB2M (20) in protein extracts from heart and liver of *Lrpprc*^+/−^ and *Lrpprc*^+/T^ mice (Fig. 4*B*), consistent with the largely normal production of mitochondrial transcripts in *in organello* transcription assays (Fig. 4*A*).

Recombinant LRPPRC has been reported to stimulate *in vitro* transcription of mtDNA in a recombinant system (18). However, we have recently reported that *in vitro* transcription reactions are very sensitive to even small alterations in salt concentrations (24), which may give the false impression that an added recombinant factor stimulates or inhibits transcription. We therefore analyzed the effects of LRPPRC on transcription while carefully controlling the exact salt concentrations. To this end, we purified recombinant LRPPRC protein to near homogeneity using affinity and ion exchange chromatography (supplemental Fig. S2). The purified LRPPRC protein was dialyzed against buffer containing 0.2 m NaCl, and the protein was also diluted in the same buffer. The *in vitro* transcription assays were performed at a final concentration of exactly 80 mm NaCl with constant amounts of POLRMT, TFB2M, and TFAM together with promoter templates containing LSP, HSP1, and HSP2 (33). Under these conditions, increasing amounts of LRPPRC did not stimulate run-off transcription from either LSP or HSP templates (Fig. 4*C* and supplemental Fig. S1*B*). We also performed EMSAs with the purified recombinant LRPPRC protein to show that it was biologically active and could bind RNA efficiently (Fig. 4*C*, *lower panel*).

We used TFAM at a concentration of 200–600 nm for the *in vitro* transcription assays, which resulted in a TFAM/mtDNA ratio similar to that observed *in vivo* (19). The authors of a previous report showing that LRPPRC stimulates transcription (18) used TFAM concentrations much lower than those observed *in vivo* (19), which potentially could have affected the outcome of their experiments. In this previous study, His-tagged LRPPRC was purified from mitochondria from transfected cell lines (18), and contamination with other mitochondrial proteins can therefore not be excluded. Given the low TFAM concentrations, even small amounts of TFAM or other transcription factors contaminating purified recombinant LRPPRC may explain the strong stimulation of transcription they observed.

**FIGURE 5.**
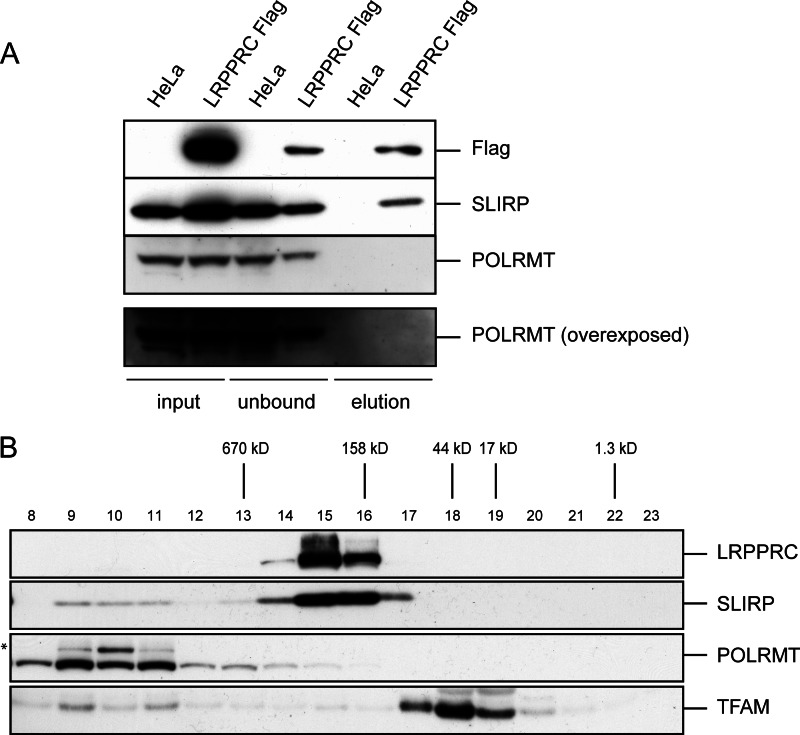
**LRPPRC does not interact with mitochondrial RNA polymerase.** *A*, co-immunoprecipitation was performed using anti-FLAG antibodies and mitochondrial extracts from HeLa cells expressing LRPPRC-FLAG. The input unbound fraction and the elution fraction obtained with FLAG peptide were analyzed by Western blotting to detect the LRPPRC-FLAG, SLIRP, and POLRMT proteins. The cell extracts analyzed were from control HeLa cells (*HeLa*) or HeLa cells transfected with LRPPRC-FLAG (*LRPPRC Flag*). *B*, size exclusion chromatography of mitochondrial extracts from wild-type HeLa cells. Western blot analysis was used to detect LRPPRC, SLIRP, POLRMT, and TFAM in the different fractions. The *asterisk* indicates an unspecific cross-reaction.

LRPPRC has been reported to directly interact with POLRMT and thereby modulate mitochondrial transcription (18). We decided to test this possibility further by using HeLa cells with doxycycline-inducible expression of human LRPPRC-FLAG. Immunoprecipitation followed by mass spectrometry revealed that LRPPRC-FLAG interacted with SLIRP, as described previously (11, 12), whereas no other partners were found (supplemental Table S1). Consistent with these results, Western blot analyses with polyclonal antibodies detected the presence of SLIRP (but not POLRMT) in the elution fraction (Fig. 5*A*). Forced expression is prone to generate protein-protein interaction artifacts, and we therefore decided to investigate whether LRPPRC interacts with other proteins under physiological *in vivo* conditions. To this end, we utilized BAC transgenic mice expressing LRPPRC-FLAG (12). This BAC transgene fully rescues the germ-line *Lrpprc* knock-out (12), showing that the expressed FLAG-tagged LRPPRC protein is fully functional. Furthermore, the expression of LRPPRC-FLAG is at levels comparable to endogenous LRPPRC expression. We performed immunoprecipitation experiments in mitochondrial extracts from liver, kidney, and heart of LRPPRC-FLAG BAC transgenic mice, followed by mass spectrometry analysis, and again identified SLIRP as an interaction partner, but not POLRMT (supplemental Table S1).

As a further means to detect a possible interaction between LRPPRC and POLRMT, we performed size exclusion chromatography on mitochondrial extracts from HeLa cells (Fig. 5*B*). LRPPRC migrated at a higher apparent molecular weight than the one predicted for LRPPRC monomers (Fig. 5*B*). There was a clear co-migration between LRPPRC and SLIRP, thus confirming previous data that these two proteins form a complex (12). However, POLRMT did not co-migrate with LRPPRC, but it rather co-migrated at a high molecular weight with TFAM. This finding could indicate interaction with TFAM and other proteins localized to the mitochondrial nucleoids (34). Interestingly, a minor portion of SLIRP co-migrated at a high molecular weight with TFAM, supporting a model in which SLIRP binds newly transcribed mRNAs close to the nucleoid, prior to processing and translation. Taken together, these data suggest that LRPPRC and POLRMT do not interact to form a stable complex.

##### LRPPRC Is Abundant in Mammalian Mitochondria

**FIGURE 6.**
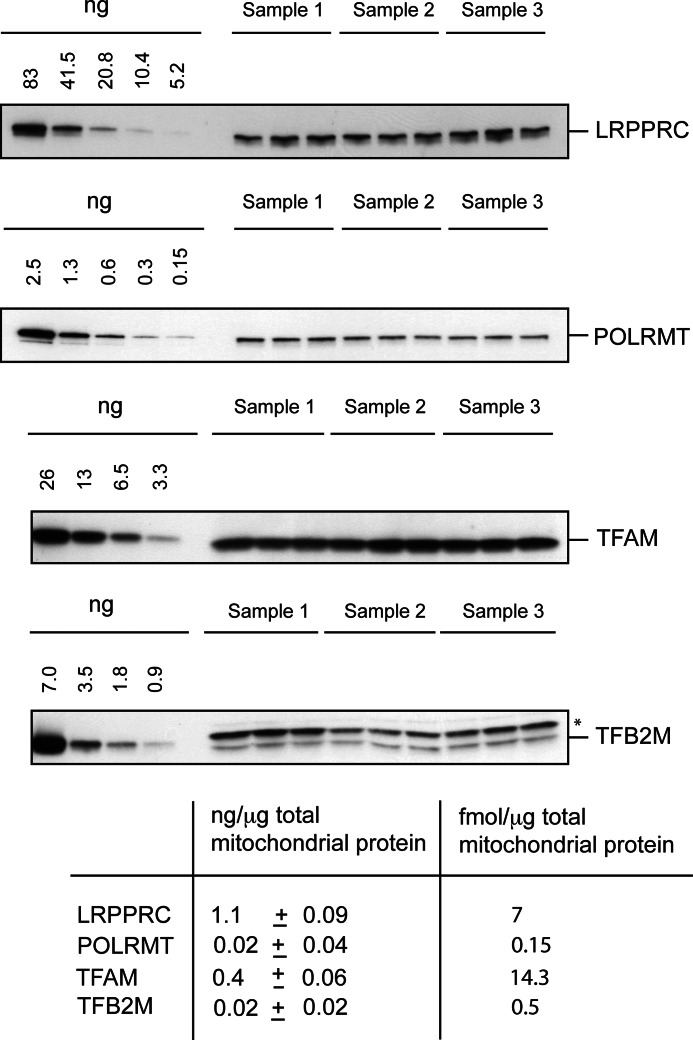
**LRPPRC is an abundant protein.** The endogenous levels of LRPPRC, POLRMT, TFAM, and TFB2M in mouse liver mitochondrial lysates were determined by Western blot analyses using purified standards of the corresponding mouse proteins. The *asterisk* indicates an unspecific cross-reacting band.

We determined the absolute levels of LRPPRC, POLRMT, TFB2M, and TFAM in mouse liver mitochondria by Western blot analyses with recombinant protein standards (Fig. 6). We found that LRPPRC was a rather abundant mitochondrial protein present at a concentration of 7 fmol/mg of total mitochondrial protein. These data are consistent with other reports that LRPPRC is abundant in human cells (13). The levels of LRPPRC are lower than the levels of TFAM, which acts as an mtDNA-packaging factor in addition to its function as a transcription factor. Interestingly, the other two components of the basal mitochondrial transcription initiation machinery, POLRMT and TFB2M, were less abundant (0.15 and 0.47 fmol/mg, respectively) (Fig. 6). The ∼50-fold higher abundance of LRPPRC in comparison with POLRMT and the ∼14-fold higher abundance in comparison with TFB2M provide another argument against a role for LRPPRC in regulation of transcription.

## DISCUSSION

LRPPRC has been reported to have several functions in the nucleus (27), cytoplasm (35), and mitochondria (11, 12). However, there are several lines of evidence suggesting that LRPPRC has predominantly a mitochondrial role. First, LRPPRC belongs to a large family of PPR motif proteins that are widespread in mitochondria and chloroplasts, where they have important roles in RNA metabolism (1). Second, cell fractionation assays and microscopy studies clearly indicate that LRPPRC is found predominantly or even exclusively in mitochondria (17). Third, genetic studies of LRPPRC in a conditional knock-out mouse model show that LRPPRC has an essential intramitochondrial role in maintaining mtDNA gene expression (12). Fourth, BSF, the fly homolog of LRPPRC, was originally reported to be a cytoplasmic protein (36), but it is located predominantly in mitochondria, where it has a critical role in regulation of fly mtDNA gene expression (31). Several of the studies in which LRPPRC has been reported to have extramitochondrial roles were based on the finding of biochemical activities in cytoplasmic or nuclear extracts (10, 16, 37). However, it cannot be excluded that these extracts have been contaminated with proteins released from broken mitochondria, and studies of extramitochondrial functions of LRPPRC should therefore be repeated under more defined conditions by using nuclear or cytoplasmic extracts that are free from contaminating mitochondrial proteins.

There are different views on the intramitochondrial role of LRPPRC. We and others have presented strong genetic data showing that LRPPRC regulates mitochondrial mRNA stability, polyadenylation, and coordination of mitochondrial translation (11–13). However, there are also reports that LRPPRC is a transcriptional activator that forms a complex with POLRMT (18, 38). On the basis of the data we have presented in this work, we find it unlikely that LRPPRC is a mitochondrial transcriptional activator because (i) moderately decreased or increased protein levels of LRPPRC *in vivo* do not affect mitochondrial transcription, (ii) immunoprecipitation experiments do not show interaction between LRPPRC and POLRMT, (iii) size exclusion chromatography gives no support for complex formation between LRPPRC and POLRMT, and (iv) purified recombinant LRPPRC does not activate mitochondrial transcription when added to a purified recombinant *in vitro* transcription system.

The steady-state levels of the ND5-Cytb precursor RNA are strongly influenced by moderate alterations of the LRPPRC protein levels *in vivo* and are decreased in heterozygous *Lrpprc* knock-out mice and increased in LRPPRC-overexpressing mice. It was recently shown that LRPPRC binds to mitochondrial precursor mRNAs, such as ATP6-COX3, tRNA^Met^-ND2, tRNA^L1^-ND1, tRNA^Val^-16 S rRNA, and tRNA^Phe^-12 S rRNA (13). It is thus possible that LRPPRC also has a function in maturation of precursor transcripts. Such a role is also suggested for BSF, the fly homolog of LRPPRC, as knockdown flies show processing aberrations in several mitochondrial transcripts (31). Taken together, our data suggest that LRPPRC has no role in mitochondrial transcriptional activation but rather functions as a regulator of mtDNA gene expression at the post-transcriptional level. We report here that the levels of a mitochondrial precursor transcript depend on LRPPRC levels, suggesting that LRPPRC may also have a role in RNA processing, in addition to the previously identified roles in regulation of mRNA stability, polyadenylation, and translational coordination in mammalian mitochondria.

## Supplementary Material

Supplemental Data

## Footnotes

2 The abbreviations used are:

PPR: pentatricopeptide repeat
BAC: bacterial artificial chromosome
Cytb: cytochrome *b*
LSP: light strand promoter
HSP: heavy strand promoter
BisTris: 2-[bis(2-hydroxyethyl)amino]-2-(hydroxymethyl)propane-1,3-diol.

## References

[1] Barkan A. Rojas M. Fujii S. Yap A. Chong Y. S. Bond C. S. Small I. (2012) A combinatorial amino acid code for RNA recognition by pentatricopeptide repeat proteins. PLoS Genet. 8, e1002910 22916040 10.1371/journal.pgen.1002910 PMC3420917

[2] Schmitz-Linneweber C. Small I. (2008) Pentatricopeptide repeat proteins: a socket set for organelle gene expression. Trends Plant Sci. 13, 663–670 19004664 10.1016/j.tplants.2008.10.001

[3] Zehrmann A. Verbitskiy D. Härtel B. Brennicke A. Takenaka M. (2011) PPR proteins network as site-specific RNA editing factors in plant organelles. RNA Biol. 8, 67–70 21289490 10.4161/rna.8.1.14298

[4] Tiranti V. Savoia A. Forti F. D'Apolito M. F. Centra M. Rocchi M. Zeviani M. (1997) Identification of the gene encoding the human mitochondrial RNA polymerase (h-mtRPOL) by cyberscreening of the Expressed Sequence Tags database. Hum. Mol. Genet. 6, 615–625 9097968 10.1093/hmg/6.4.615

[5] Rackham O. Davies S. M. K. Shearwood A.-M. J. Hamilton K. L. Whelan J. Filipovska A. (2009) Pentatricopeptide repeat domain protein 1 lowers the levels of mitochondrial leucine tRNAs in cells. Nucleic Acids Res. 37, 5859–5867 19651879 10.1093/nar/gkp627 PMC2761286

[6] Xu F. Ackerley C. Maj M. C. Addis J. B. L. Levandovskiy V. Lee J. Mackay N. Cameron J. M. Robinson B. H. (2008) Disruption of a mitochondrial RNA-binding protein gene results in decreased cytochrome *b* expression and a marked reduction in ubiquinol-cytochrome *c* reductase activity in mouse heart mitochondria. Biochem. J. 416, 15–26 18729827 10.1042/BJ20080847

[7] Davies S. M. K. Rackham O. Shearwood A.-M. J. Hamilton K. L. Narsai R. Whelan J. Filipovska A. (2009) Pentatricopeptide repeat domain protein 3 associates with the mitochondrial small ribosomal subunit and regulates translation. FEBS Lett. 583, 1853–1858 19427859 10.1016/j.febslet.2009.04.048

[8] Davies S. M. K. Lopez Sanchez M. I. G. Narsai R. Shearwood A.-M. J. Razif M. F. M. Small I. D. Whelan J. Rackham O. Filipovska A. (2012) MRPS27 is a pentatricopeptide repeat domain protein required for the translation of mitochondrially encoded proteins. FEBS Lett. 586, 3555–3561 22841715 10.1016/j.febslet.2012.07.043

[9] Holzmann J. Frank P. Löffler E. Bennett K. L. Gerner C. Rossmanith W. (2008) RNase P without RNA: identification and functional reconstitution of the human mitochondrial tRNA processing enzyme. Cell 135, 462–474 18984158 10.1016/j.cell.2008.09.013

[10] Mili S. Piñol-Roma S. (2003) LRP130, a pentatricopeptide motif protein with a noncanonical RNA-binding domain, is bound *in vivo* to mitochondrial and nuclear RNAs. Mol. Cell. Biol. 23, 4972–4982 12832482 10.1128/MCB.23.14.4972-4982.2003 PMC162214

[11] Sasarman F. Brunel-Guitton C. Antonicka H. Wai T. Shoubridge E. A., and LSFC Consortium (2010) LRPPRC and SLIRP interact in a ribonucleoprotein complex that regulates post-transcriptional gene expression in mitochondria. Mol. Biol. Cell 21, 1315–1323 20200222 10.1091/mbc.E10-01-0047 PMC2854090

[12] Ruzzenente B. Metodiev M. D. Wredenberg A. Bratic A. Park C. B. Cámara Y. Milenkovic D. Zickermann V. Wibom R. Hultenby K. Erdjument-Bromage H. Tempst P. Brandt U. Stewart J. B. Gustafsson C. M. Larsson N.-G. (2012) LRPPRC is necessary for polyadenylation and coordination of translation of mitochondrial mRNAs. EMBO J. 31, 443–456 22045337 10.1038/emboj.2011.392 PMC3261557

[13] Chujo T. Ohira T. Sakaguchi Y. Goshima N. Nomura N. Nagao A. Suzuki T. (2012) LRPPRC/SLIRP suppresses PNPase-mediated mRNA decay and promotes polyadenylation in human mitochondria. Nucleic Acids Res. 40, 8033–8047 22661577 10.1093/nar/gks506 PMC3439899

[14] Xu F. Morin C. Mitchell G. Ackerley C. Robinson B. H. (2004) The role of the *LRPPRC* (leucine-rich pentatricopeptide repeat cassette) gene in cytochrome oxidase assembly: mutation causes lowered levels of COX (cytochrome *c* oxidase) I and COX III mRNA. Biochem. J. 382, 331–336 15139850 10.1042/BJ20040469 PMC1133946

[15] Topisirovic I. Siddiqui N. Orolicki S. Skrabanek L. A. Tremblay M. Hoang T. Borden K. L. B. (2009) Stability of eukaryotic translation initiation factor 4E mRNA is regulated by HuR, and this activity is dysregulated in cancer. Mol. Cell. Biol. 29, 1152–1162 19114552 10.1128/MCB.01532-08 PMC2643828

[16] Cooper M. P. Uldry M. Kajimura S. Arany Z. Spiegelman B. M. (2008) Modulation of PGC-1 coactivator pathways in brown fat differentiation through LRP130. J. Biol. Chem. 283, 31960–31967 18728005 10.1074/jbc.M805431200 PMC2581541

[17] Sterky F. H. Ruzzenente B. Gustafsson C. M. Samuelsson T. Larsson N.-G. (2010) LRPPRC is a mitochondrial matrix protein that is conserved in metazoans. Biochem. Biophys. Res. Commun. 398, 759–764 20633537 10.1016/j.bbrc.2010.07.019

[18] Liu L. Sanosaka M. Lei S. Bestwick M. L. Frey J. H. Jr. Surovtseva Y. V. Shadel G. S. Cooper M. P. (2011) LRP130 protein remodels mitochondria and stimulates fatty acid oxidation. J. Biol. Chem. 286, 41253–41264 21971050 10.1074/jbc.M111.276121 PMC3308838

[19] Ekstrand M. I. Falkenberg M. Rantanen A. Park C. B. Gaspari M. Hultenby K. Rustin P. Gustafsson C. M. Larsson N.-G. (2004) Mitochondrial transcription factor A regulates mtDNA copy number in mammals. Hum. Mol. Genet. 13, 935–944 15016765 10.1093/hmg/ddh109

[20] Falkenberg M. Gaspari M. Rantanen A. Trifunovic A. Larsson N.-G. Gustafsson C. M. (2002) Mitochondrial transcription factors B1 and B2 activate transcription of human mtDNA. Nat. Genet. 31, 289–294 12068295 10.1038/ng909

[21] Spåhr H. Habermann B. Gustafsson C. M. Larsson N.-G. Hällberg B. M. (2012) Structure of the human MTERF4-NSUN4 protein complex that regulates mitochondrial ribosome biogenesis. Proc. Natl. Acad. Sci. U.S.A. 109, 15253–15258 22949673 10.1073/pnas.1210688109 PMC3458362

[22] Gaspari M. Falkenberg M. Larsson N.-G. Gustafsson C. M. (2004) The mitochondrial RNA polymerase contributes critically to promoter specificity in mammalian cells. EMBO J. 23, 4606–4614 15526033 10.1038/sj.emboj.7600465 PMC533051

[23] Wong T. S. Rajagopalan S. Freund S. M. Rutherford T. J. Andreeva A. Townsley F. M. Petrovich M. Fersht A. R. (2009) Biophysical characterizations of human mitochondrial transcription factor A and its binding to tumor suppressor p53. Nucleic Acids Res. 37, 6765–6783 19755502 10.1093/nar/gkp750 PMC2777442

[24] Shi Y. Dierckx A. Wanrooij P. H. Wanrooij S. Larsson N.-G. Wilhelmsson L. M. Falkenberg M. Gustafsson C. M. (2012) Mammalian transcription factor A is a core component of the mitochondrial transcription machinery. Proc. Natl. Acad. Sci. U.S.A. 109, 16510–16515 23012404 10.1073/pnas.1119738109 PMC3478657

[25] Bogenhagen D. F. Rousseau D. Burke S. (2008) The layered structure of human mitochondrial DNA nucleoids. J. Biol. Chem. 283, 3665–3675 18063578 10.1074/jbc.M708444200

[26] Larsson N.-G. (2010) Somatic mitochondrial DNA mutations in mammalian aging. Annu. Rev. Biochem. 79, 683–706 20350166 10.1146/annurev-biochem-060408-093701

[27] Cooper M. P. Qu L. Rohas L. M. Lin J. Yang W. Erdjument-Bromage H. Tempst P. Spiegelman B. M. (2006) Defects in energy homeostasis in Leigh syndrome French Canadian variant through PGC-1α/LRP130 complex. Genes Dev. 20, 2996–3009 17050673 10.1101/gad.1483906 PMC1620022

[28] Park C. B. Asin-Cayuela J. Cámara Y. Shi Y. Pellegrini M. Gaspari M. Wibom R. Hultenby K. Erdjument-Bromage H. Tempst P. Falkenberg M Gustafsson C. M. Larsson N.-G. (2007) MTERF3 is a negative regulator of mammalian mtDNA transcription. Cell 130, 273–285 17662942 10.1016/j.cell.2007.05.046

[29] Metodiev M. D. Lesko N. Park C. B. Cámara Y. Shi Y. Wibom R. Hultenby K. Gustafsson C. M. Larsson N.-G. (2009) Methylation of 12S rRNA is necessary for *in vivo* stability of the small subunit of the mammalian mitochondrial ribosome. Cell Metab. 9, 386–397 19356719 10.1016/j.cmet.2009.03.001

[30] Cámara Y. Asin-Cayuela J. Park C. B. Metodiev M. D. Shi Y. Ruzzenente B. Kukat C. Habermann B. Wibom R. Hultenby K. Franz T Erdjument-Bromage H. Tempst P. Hallberg B. M. Gustafsson C. M. Larsson N.-G. (2011) MTERF4 regulates translation by targeting the methyltransferase NSUN4 to the mammalian mitochondrial ribosome. Cell Metab. 13, 527–539 21531335 10.1016/j.cmet.2011.04.002

[31] Bratic A. Wredenberg A. Grönke S. Stewart J. B. Mourier A. Ruzzenente B. Kukat C. Wibom R. Habermann B. Partridge L. Larsson N.-G. (2011) The bicoid stability factor controls polyadenylation and expression of specific mitochondrial mRNAs in *Drosophila melanogaster*. PLoS Genet. 7, e1002324 22022283 10.1371/journal.pgen.1002324 PMC3192837

[32] Parisi M. A. Clayton D. A. (1991) Similarity of human mitochondrial transcription factor 1 to high mobility group proteins. Science 252, 965–969 2035027 10.1126/science.2035027

[33] Falkenberg M. Larsson N.-G. Gustafsson C. M. (2007) DNA replication and transcription in mammalian mitochondria. Annu. Rev. Biochem. 76, 679–699 17408359 10.1146/annurev.biochem.76.060305.152028

[34] Hällberg B. M. Larsson N.-G. (2011) TFAM forces mtDNA to make a U-turn. Nat. Struct. Mol. Biol. 18, 1179–1181 22056802 10.1038/nsmb.2167

[35] Liu L. McKeehan W. L. (2002) Sequence analysis of LRPPRC and its SEC1 domain interaction partners suggests roles in cytoskeletal organization, vesicular trafficking, nucleocytosolic shuttling, and chromosome activity. Genomics 79, 124–136 11827465 10.1006/geno.2001.6679 PMC3241999

[36] Mancebo R. Zhou X. Shillinglaw W. Henzel W. Macdonald P. M. (2001) BSF binds specifically to the bicoid mRNA 3′ untranslated region and contributes to stabilization of bicoid mRNA. Mol. Cell. Biol. 21, 3462–3471 11313472 10.1128/MCB.21.10.3462-3471.2001 PMC100268

[37] Mili S. Shu H. J. Zhao Y. Piñol-Roma S. (2001) Distinct RNP complexes of shuttling hnRNP proteins with pre-mRNA and mRNA: candidate intermediates in formation and export of mRNA. Mol. Cell. Biol. 21, 7307–7319 11585913 10.1128/MCB.21.21.7307-7319.2001 PMC99905

[38] Sondheimer N. Fang J.-K. Polyak E. Falk M. J. Avadhani N. G. (2010) Leucine-rich pentatricopeptide-repeat containing protein regulates mitochondrial transcription. Biochemistry 49, 7467–7473 20677761 10.1021/bi1008479 PMC2932791

