# Supplementary material for: The Leucine-rich Pentatricopeptide Repeat-containing Protein (LRPPRC) Does Not Activate Transcription in Mammalian Mitochondria
Source: J Biol Chem. 2013 Apr 18;288(22):15510–9. doi: 10.1074/jbc.M113.471649 (PMC3668712; doi:10.1074/jbc.M113.471649)
Supplement: Supplemental Data [file supp_288_22_15510__index.html]

The leucine-rich pentatricopeptide repeat containing (LRPPRC) protein does not activate transcription in mammalian mitochondria — The Leucine-rich Pentatricopeptide Repeat-containing Protein (LRPPRC) Does Not Activate Transcription in Mammalian Mitochondria — LRPPRC Does Not Activate mtDNA Transcription — Supplemental Data 

# The Leucine-rich Pentatricopeptide Repeat-containing Protein (LRPPRC) Does Not Activate Transcription in Mammalian Mitochondria

## Supplemental Data

**Files in this Data Supplement:**

- Supplemental Data (.pdf, 2.7 MB) - compiled supplemental data to the manuscript
