# Supplementary material for: The Leucine-rich Pentatricopeptide Repeat-containing Protein (LRPPRC) Does Not Activate Transcription in Mammalian Mitochondria
Source: J Biol Chem. 2013 Apr 18;288(22):15510–9. doi: 10.1074/jbc.M113.471649 (PMC3668712; doi:10.1074/jbc.M113.471649)
Supplement: Supplemental Data [file supp_M113.471649_jbc.M113.471649-1.pdf]

SUPPLEMENTAL DATA

FIGURE S1: Quantification of gels. A**.** Quantification of areas a – d of Figure 4A. B. Quantification of HSP and LSP run-off transcripts from n=4 *in vitro* transcription experiments. For LSP the values for run-off (RO) and pre-terminated transcript at CSBII (PT) were used. The relative amount 1 indicates transcript levels in the absence of LRPPRC. Error bars indicate SD.

FIGURE S2: Coomassie staining. Coomassie staining of a gel showing recombinant human LRPPRC-6xHis-Tag purified from Sf9 cells as described in the material and methods section.

SUPPLEMENTAL EXPERIMENTAL PROCEDURES

Protein identification with LC-MS/MS after Immunoprecipitation

50ul of 25mM ammonium bicarbonate was added to each pellet. Vortex to dissolve the protein pellet completely. 3ul of 1% RapiGest (Waters Corporation, Milford, USA) was added for denaturing the proteins. The protein solution was incubated at 80°C for 10 min on a Thermo-mixer. After addition of 2.5µl aliquot of 50mM DTT (Sigma–Aldrich) the solution was heated at 60°C for 15 min. The protein solution was then cooled down to room temperature and centrifuged. After the addition of 2.5µl aliquot of 150mM iodoacetamide (Sigma–Aldrich), the solution was stored in the dark at room temperature for 30 min. The tryptic digestion was performed by adding Trypsin Gold mass spectrometry grade (Promega, Madison, MI, USA) at a 1:50 (w/w) ratio and incubating at 37oC overnight. 1µl of 37% HCL was added to adjust the pH below 2. After being vortexed and centrifuged at 13000xg for 30 min, the supernatant was collected and transferred to a clean microcentrifuge tube. The protein digest was dried in Speed-Vac and re-suspended with 10ul of 0.1% of formic acid.

Samples were analyzed by LC-ESI-MS/MS with an Amazon ion trap ETD (Bruker, Bremen, Germany) coupled to an Aquity nanoUPLC with sample manager (Waters, Manchester, UK) using a 40 min gradient (3% to 55% ACN) at 300 nL/min flow, 15 min washing step (95% ACN) followed by re-equilibration for 20 min (3% ACN). The ion trap was operated in positive MS mode at enhanced resolution speed of 8100 m/z/s. ICC target was set to 400000 and the maximum accumulation time to 50 ms.

Scan range was m/z 300-1300. The source capillary was operated at -4500 V and the end plate offset was -500 V. The nebulizer pressure was 15 PSI, dry gas flow 4.0 L/min and the dry gas temperature 200°C. MS/MS parameters: number of precursors were 10, threshold absolute 25000, MS/MS fragmentation amplitude was 0.8 V, isolation width 2.5 m/z, scan speed 32500 m/z/s, scan range m/z 100-2400, ICC target 100000, maximum accumulation time 100 ms, precursor was excluded after one spectra and released after 0.17 min. The raw data were processed with DataAnalysis (Version 4.0 SP 2, Bruker, Bremen Germany). The processed data were imported into ProteinScape 2.1.0577 (Bruker, Bremen, Germany) and the extracted MS/MS data were submitted to an in-house MASCOT server (version 2.3, Matrix Science, London, UK). Proteins were identified by searching the peptide lists against Knowledgebase 2012_11 (538585 sequences and 191240774 residues).

The following parameters were used: taxonomy Mus musculus (16580 sequences); Enzyme: trypsin; Max Missed Cleavages: 1; Fixed modifications: carbamidomethyl (C); Variable modifications : oxidation (M); Peptide Mass Tolerance : ±0.8 Da: Fragment Mass Tolerance: ± 0.8 Da. ((1))

Protein identification and quantification with LC-MS/MS

Samples were diluted with 100mM ammonium bicarbonate to gain a concentration of about 200ng/µl according to Bradford method. Tryptic digestion was performed by adding Trypsin Gold mass spectrometry grade (Promega, Madison, MI, USA) at a 1:50 (w/w) ratio and incubation at 37°C overnight.

Protein identification and quantification were performed with a Xevo Q-Tof (Waters Corporation, Milford, USA) coupled with a nanoACQUITY UPLC™ (Waters Corporation, Milford USA). The digest was 10 fold diluted with 0.1% of formic acid. 1µl of standard Alcohol dehydrogenase 1 (ADH) tryptic digest (50fmol/µl) (Waters Corporation, Milford, USA) was added to 0.5µl of the sample digest. 1.5µl of the digest mixer was loaded into a C18 trap column of 180 µm X 20 mm with 10µl/min of 3% of solvent A (0.1% Formic Acid) for 2 min. The digest was then separated and eluted with an analytical column of 75 µm x 150mm C18 BEH 1.7 µm (Waters Corporation, Milford USA). The gradient was 3% to 35% of acetonitrile in 0.1% formic acid over 10min at a flow rate of 400nl/min. The Xevo Q-Tof was operated in LC/MS^E mode over the m/z range of 50-1800 in nano electrospray mode. The capillary,

sample cone, extraction cone and collision energy were 3.3kV, 25.0V, 2.0V, and 6.0V respectively. During elevated energy scan, the collision energy was ramped from 15V to 35V. Glu-fibrinopeptide B of m/z 785.84 was used as Lock Mass for mass correction. At least 3 replicates of one sample were analyzed.
Data was collected using MassLynx™ 4.1 and processed and searched using ProteinLynx™ Global Server 2.5.2. (Waters Corporation, Milford USA). Following parameters were used for database search: enzyme "trypsin", minimal fragments ion per peptide matched ''2'', minimal fragments ion per protein matched ''7'', missed cleavages ''1'', variable modification ''oxidation Methionine'', peptide tolerance ''automatic'', fragment tolerance'' automatic'', false positive rate ''4%''. Calibration protein P00330 (ADH1_YEAST), calibration protein concentration 50fmol. The mouse database was from Uniprot release knowledgebase_2012_11.

References

1. Franz,T. and Li,X. (2012) The OASIS® HLB μElution plate as a one-step platform for manual high-throughput in-gel digestion of proteins and peptide desalting. *Proteomics*, **12**, 2487–2492.

A

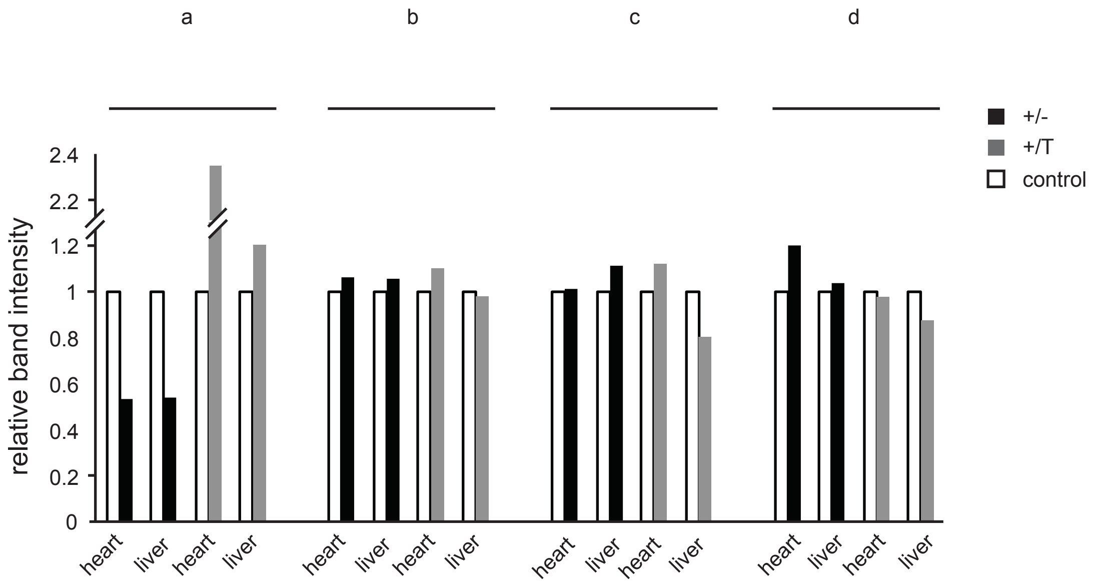

B

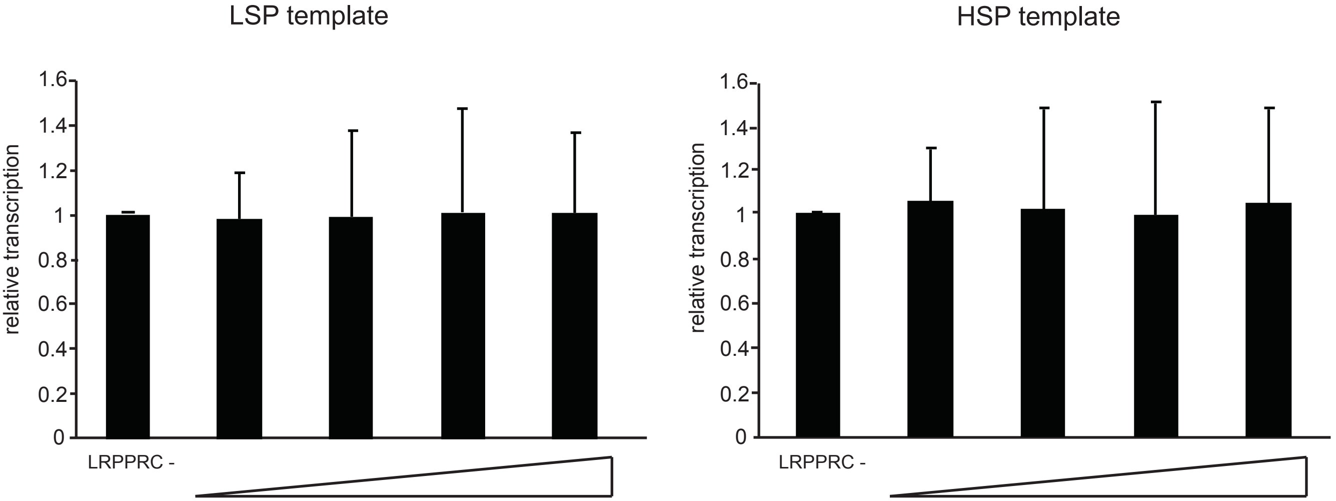

Suppl. Figure 1

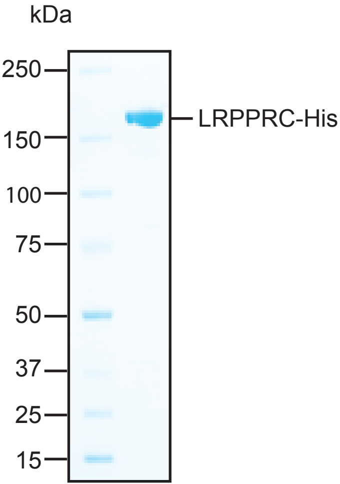

Suppl. Figure 2

Table 1: LRPPRC Immunoprecipitation- Mass spectrometry results.
SC= Sequence Score, emPAI=Exponentially Modified Protein Abundance Index

	Gene	UNIPROT	PROT NAME	PEPTIDES	SCORE	SC	emPAI
HEART MITOCHONDRIA	LRPPRC_MOUSE	Q6PB66	Leucine-rich PPR motif-containing protein	12	1273.7	10.1	0.16
	SLIRP_MOUSE	Q9D8T7	SRA stem-loop-interacting RNA-binding protein	1	107.3	10.7	0.27
	K1C10_MOUSE	P02535	Keratin, type I cytoskeletal 10	2	153.2	2.8	0.06
	K2C1_MOUSE	P04104	Keratin, type II cytoskeletal 1	1	99.3	1.9	0.05
KIDNEY MITOCHONDRIA	LRPPRC_MOUSE	Q6PB66	Leucine-rich PPR motif-containing protein	40	1779.9	30.9	1.06
	SLIRP_MOUSE	Q9D8T7	SRA stem-loop-interacting RNA-binding protein	6	279.2	49.1	1.63
	MCCA_MOUSE	Q99MR8	Methylcrotonoyl-CoA carboxylase subunit alpha	5	294.6	8.5	0.28
	MCCB_MOUSE	Q3ULD5	Methylcrotonoyl-CoA carboxylase beta chain	11	504.4	20.1	0.6
	ECHB_MOUSE	Q99JY0	Trifunctional enzyme subunit beta	1	62.0	1.9	0.06
	ECHP_MOUSE	Q9DBM2	Peroxisomal bifunctional enzyme	1	54.7	2.1	0.04
LIVER MITOCHONDRIA	LRPPRC_MOUSE	Q6PB66	Leucine-rich PPR motif-containing protein	40	1797.6	30.2	1.19
	SLIRP_MOUSE	Q9D8T7	SRA stem-loop-interacting RNA-binding protein	8	405.1	59.8	4.42
	PPA5_MOUSE	Q05117	Tartrate-resistant acid phosphatase type 5	2	94.6	9.5	0.19
	DHE3_MOUSE	P26443	Glutamate dehydrogenase 1	2	93.2	5.9	0.05
	SPA3N_MOUSE	Q91WP6	Serine protease inhibitor A3N	1	88.8	4.5	0.07
	PPIB_MOUSE	P24369	Peptidyl-prolyl cis-trans isomerase B	1	56.4	6	0.14
	ODO2_MOUSE	Q9D2G2	Dihydrolipoyllysine-residue succinyltransferase component of 2-oxoglutarate dehydrogenase complex	1	38.6	1.3	0.07
	DLDH_MOUSE	O08749	Dihydrolipoyl dehydrogenase	1	35.3	2.8	0.06
HELA CELLS	LRPPRC_HUMAN	P42704	Leucine-rich PPR motif-containing protein	37	1686.4	31.2	0.98
	SLIRP_HUMAN	Q9GZT3	SRA stem-loop-interacting RNA-binding protein	8	390.5	44.3	1.7
	DOC11_HUMAN	Q5JSL3	Dedicator of cytokinesis protein 11	2	75.7	6.8	0.5
	PGRC1_HUMAN	O00264	Membrane-associated progesterone receptor component 1	1	80.1	5.8	0.04
	ACTBL_HUMAN	Q562R1	Beta-actin-like protein 2	2	49.0	2.1	0.06

Suppl. Table 1
